# High stretch induces endothelial dysfunction accompanied by oxidative stress and actin remodeling in human saphenous vein endothelial cells

**DOI:** 10.1038/s41598-021-93081-3

**Published:** 2021-06-29

**Authors:** T. Girão-Silva, M. H. Fonseca-Alaniz, J. C. Ribeiro-Silva, J. Lee, N. P. Patil, L. A. Dallan, A. B. Baker, M. C. Harmsen, J. E. Krieger, A. A. Miyakawa

**Affiliations:** 1 Laboratory of Genetics and Molecular Cardiology, Heart Institute (InCor), University of Sao Paulo Medical School, Av. Dr. Eneas de Carvalho Aguiar, 44-Bloco II, 10 Andar, São Paulo, SP 05403-000 Brazil; 2 Department of Biomedical Engineering, Institute for Cellular and Molecular Biology, Institute for Biomaterials, Drug Delivery and Regenerative Medicine, Institute for Computational Engineering and Sciences, University of Texas at Austin, Austin, TX USA; 3 Laboratory for Cardiovascular Regenerative Medicine Research Group (CAVAREM), Department of Pathology and Medical Biology, University Medical Center Groningen, University of Groningen, Groningen, the Netherlands

**Keywords:** Cell biology, Cell signalling, Cardiology, Cardiovascular biology

## Abstract

The rate of the remodeling of the arterialized saphenous vein conduit limits the outcomes of coronary artery bypass graft surgery (CABG), which may be influenced by endothelial dysfunction. We tested the hypothesis that high stretch (HS) induces human saphenous vein endothelial cell (hSVEC) dysfunction and examined candidate underlying mechanisms. Our results showed that in vitro HS reduces NO bioavailability, increases inflammatory adhesion molecule expression (E-selectin and VCAM1) and THP-1 cell adhesion. HS decreases F-actin in hSVECs, but not in human arterial endothelial cells, and is accompanied by G-actin and cofilin’s nuclear shuttling and increased reactive oxidative species (ROS). Pre-treatment with the broad-acting antioxidant N-acetylcysteine (NAC) supported this observation and diminished stretch-induced actin remodeling and inflammatory adhesive molecule expression. Altogether, we provide evidence that increased oxidative stress and actin cytoskeleton remodeling play a role in HS-induced saphenous vein endothelial cell dysfunction, which may contribute to predisposing saphenous vein graft to failure.

## Introduction

Saphenous veins (SVs) are widely used in coronary artery bypass graft (CABG) surgeries to revascularize ischemic hearts due to their length and the ease with which they can be obtained surgically, despite their reduced patency compared to arterial conduits^1,2^. Failure occurs acutely (within the first month) due to thrombosis associated with SV handling and surgical anastomosis, ischemia/reperfusion and endothelium denudation, whereas late graft occlusion arises due to progressive intimal hyperplasia, excessive extracellular matrix production and atherosclerotic plaque development^1–3^. Improvements in surgical procedures and postoperative medication have mitigated some the early graft failure, but adverse long-term outcomes remain a critical problem, as 50–60% of patients develop vein graft failure within 10 years^1,2^.

The SV graft wall is exposed to an abrupt hemodynamic overload and must remodel to function as an artery, a process known as arterialization, which stabilizes mechanical forces and involves muscle cell (SMC) proliferation and extracellular matrix production^1,2^. This SV remodeling capacity under different hemodynamic conditions is underscored by the fact that arterialization is induced by interposing a vein segment in an arterial bed, followed by reverse remodeling when the vessel is placed back in the venous condition^3^. The problem is that the remodeling process may exceed what is required to stabilize the mechanical forces and result in vessel lumen narrowing and late graft failure^1,2^.

The endothelium plays a critical role in the vascular homeostasis and endothelial dysfunction is an important common underlying factor among cardiovascular diseases^3–5^. In the SV grafting, there is a great increase in shear force and stretch, that elicits intracellular downstream signaling to alter endothelial cell (EC) function^1–5^. It has been demonstrated that sudden increase of shear stress induces inflammatory EC phenotype and contributes to vein graft failure^6,7^. The impact of shear stress in EC is widely studied while less is known about the stretch effects in venous EC^6–8^. Indeed, circumferential stretch is a critical factor for neointima hyperplasia at vein graft disease^9,10^. External stents placed at the vein graft, to limit the vessel distention, attenuates intimal hyperplasia, prevents vascular cell injury and inhibits the augmented VCAM and ICAM1 expression induced by arterial stress^9,11,12^. Of note, stenting avoids the vein circumferential stretch while shear stress increases suggesting that endothelial dysfunction on SV graft relies more on increased cyclic stretch than on shear stress^9^. However, the underlying cellular mechanisms of increased cyclic stretch on human saphenous vein endothelial cell (hSVEC) phenotype remains largely unclear.

Here, we used different levels of stretch on hSVECs up to 48 h to assess whether and how it may induce endothelial dysfunction. Our data demonstrate that sustained high stretch (HS) induces endothelial dysfunction and actin filament remodeling mediated by reactive oxygen species (ROS).

## Results

### Sustained high stretch causes endothelial dysfunction

**Figure 1:**
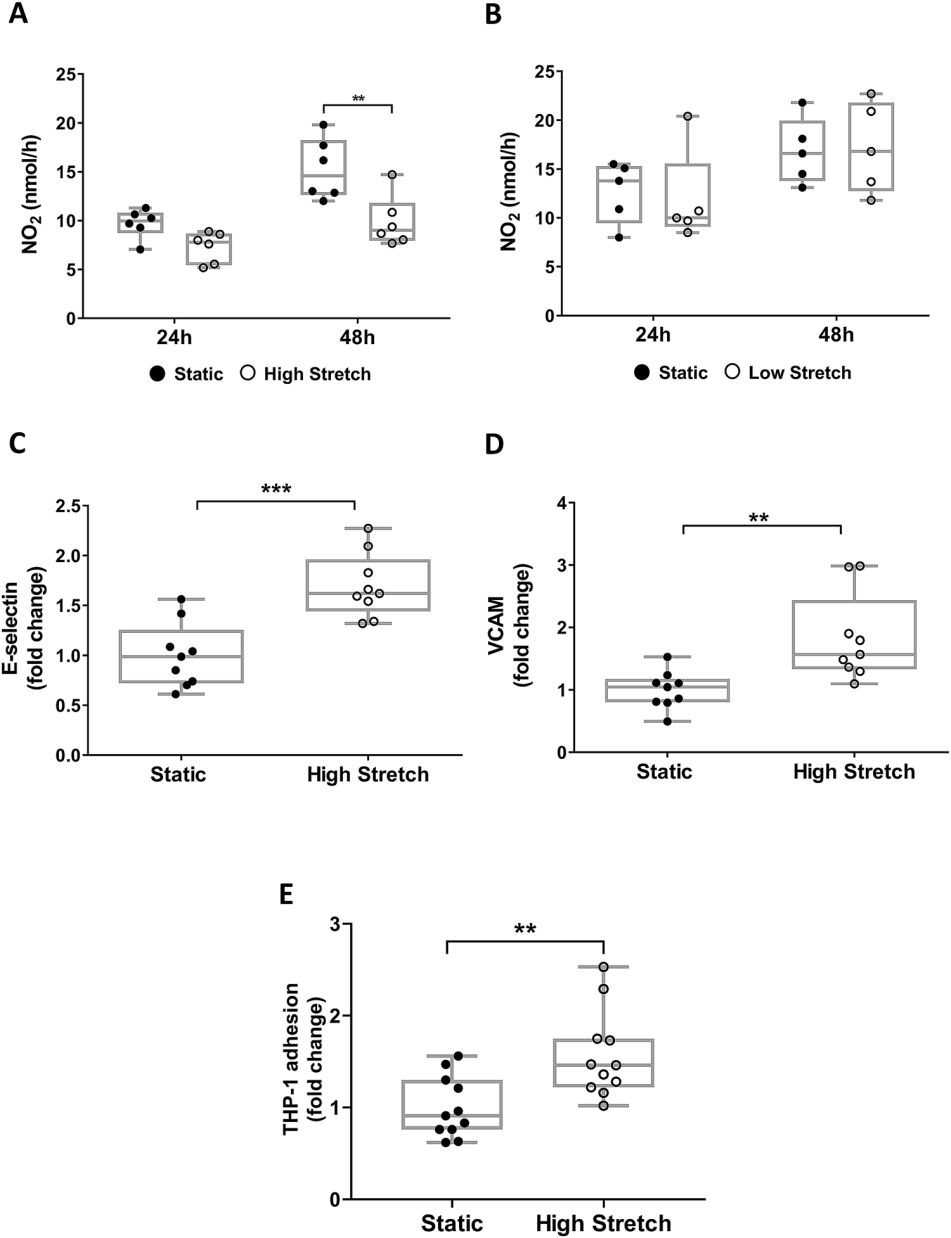
High stretch promotes hSVECs dysfunction. Nitric oxide was measured by its metabolite Nitrite (NO_2_). Rate of NO_2_ amount following high (**A**) and low (**B**) stretch at the indicated time of stimulus, N = 6. Flow cytometry analysis was used for pro-inflammatory markers and monocyte adhesion detection. E-selectin (**C**) and VCAM 1 expression (**D**) after 48 h of hSVECs at high stretch, N = 9. The data are represented as mean ± SEM of at least 2 independent experiments with cells obtained from different donors. THP-1 adhesion on hSVECs after 48 h of high stretch (**E**), N = 11. The data are represented as mean ± SEM of 3 independent experiments with cells obtained from different donors. Two-way ANOVA with Tukey's multiple comparisons test **(A,B)** and unpaired t-test **(C–E)** was used for statistical analysis,* indicates p < 0.05, ** indicates p < 0.01 and *** indicates p < 0.001. Flow cytometry data are plotted as fold change related to static control group and representative flow cytometry plots are shown in the Supplementary Information.

Given the important effects of mechanical forces on EC structure and function, we exposed hSVECs to low stretch (LS, venous) and HS (arterial) conditions to mimic abrupt hemodynamic stress during vein arterialization. HS reduced the NO release at 48 h compared to static control (Fig. 1A) while there were no changes induced by LS (Fig. 1B). The synthesis and release of NO by ECs is modulated by hemodynamic forces (flow and cyclic strain) to regulate vascular tone and blood flow, and the HS-induced reduction of NO indicates endothelial dysfunction^13,14^. Indeed, we observed an increase in the surface expression of inflammatory markers E-selectin (Fig. 1C) and VCAM 1 (Fig. 1D), and an augmented adhesion of monocytes after sustained HS (Fig. 1E) further confirming the endothelial dysfunction phenotype.

**Figure 2:**
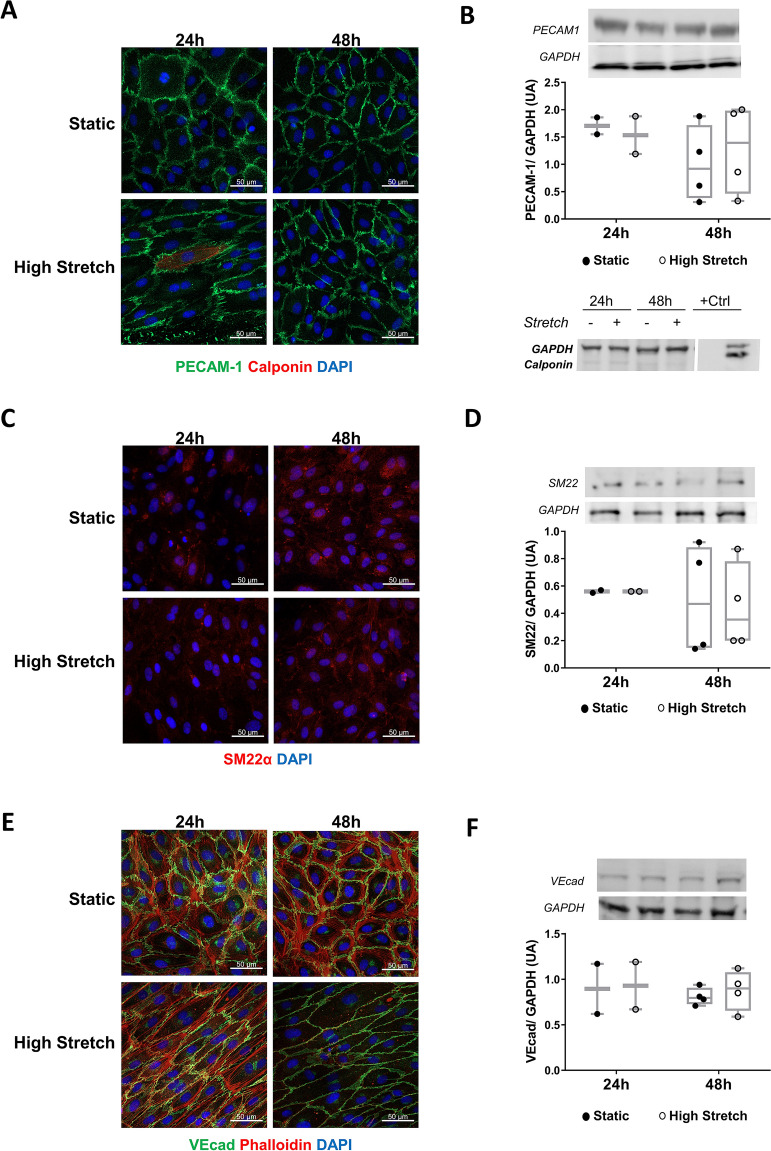
High stretch does not induce endothelial-to-mesenchymal transition (EndMT) in hSVECs. Cells were submitted to high stretch up to 48 h and analyzed for endothelial and mesenchymal markers by immunostaining and western blot. Confocal images for PECAM-1 (green) and Calponin (red) (**A**), western blot data for PECAM-1 N = 2–4 and Calponin N = 3 (**B**). As Calponin was not detected in hSVECs, smooth muscle cell protein was used as positive control. SM22α analyzed by confocal images (**C**) and by western blot N = 2–4 (**D**). Confocal image for VE-cadherin (green) and F-actin (red) (**E**), western blot data for VE-cadherin N = 2–4 (**F**). All confocal experiments were also stained for nucleus with DAPI (blue), magnification: 400 × and scale: 50 µm (white bar). All western blot experiments were normalized by GAPDH blotted in the same gel as the target protein (see full-length gels and blots in the Supplementary Information).

In response to pathological triggers, EC may adopt a mesenchymal phenotype via EndMT in several diseases, including evidence related to vein graft disease^15–17^. As sustained HS lead to endothelial dysfunction, we tested whether it would also induce a mesenchymal phenotype. The hSVECs can undergo phenotypical switching, since TGF-β_2_ and IL-1β treatment promptly stimulate the transition to mesenchymal phenotype (See Suplementary Fig. S1 online), yet HS failed to induce EndMT in hSVECs (Fig. 2). We observed no reduction in the endothelial markers PECAM-1 (Fig. 2A,B) and VE-cadherin (Fig. 2E,F). In addition, there was no increase in the expression of the mesenchymal markers calponin (Fig. 2A,B) and SM22α (Fig. 2C,D) and HS neither accelerated nor potentiated growth factor-induced EndMT (See Suplementary Fig. S2 online). In contrast, we observed reduction in F-actin staining following 48 h of stretching (Fig. 2E). Given the crucial effects of actin cytoskeleton to EC structure and function, we further investigated the impact of HS on F-actin pattern and EC dysfunction.

### HS remodels actin filaments in venous ECs but not in arterial ECs

**Figure 3:**
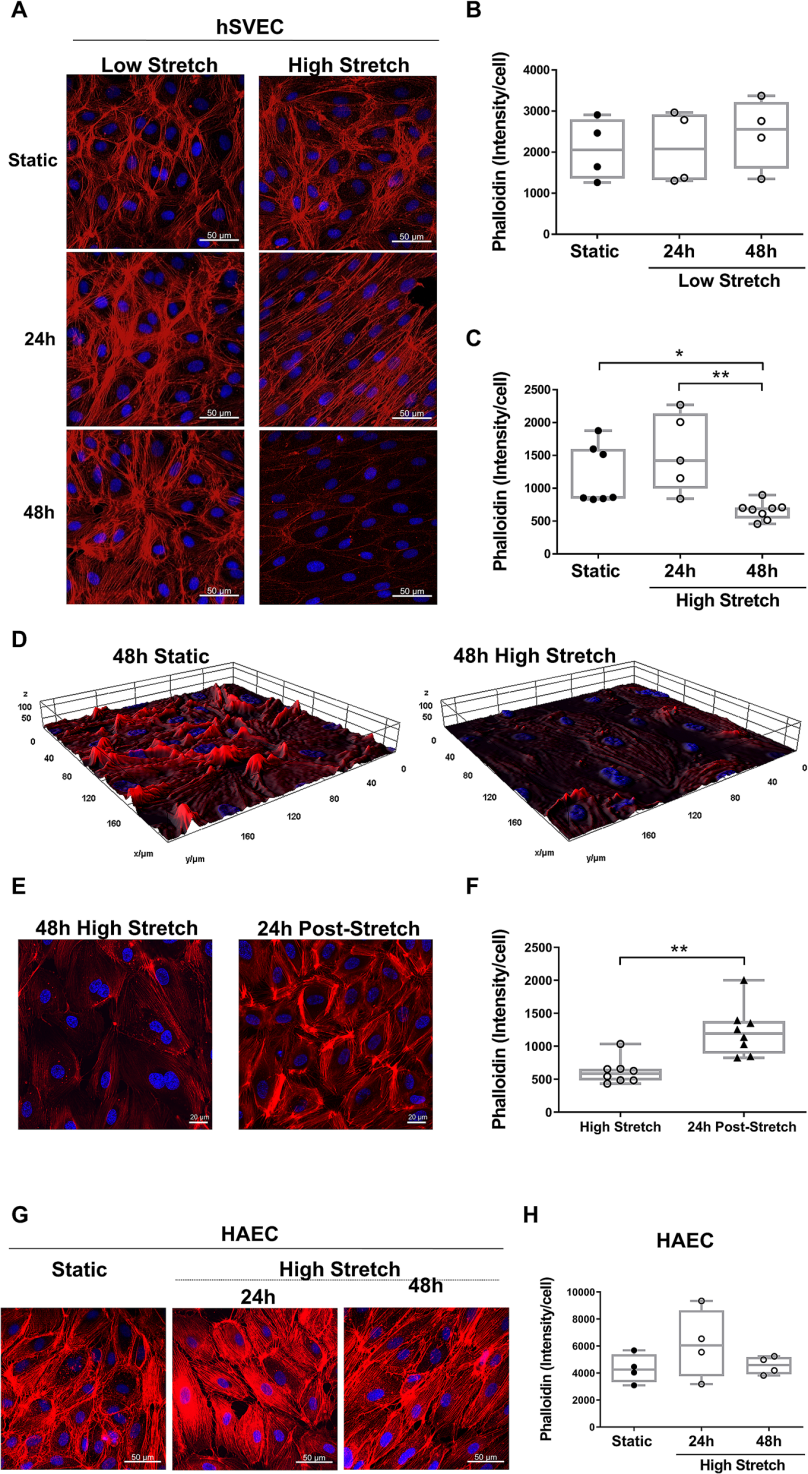
Actin filaments staining is reduced by high stretch in hSVECs but not in HAEC and this effect reversed after 24 h in static conditions. Panel with confocal images of F-actin stained with phalloidin (red) from hSVECs submitted to different stretch level up to 48 h (**A**). Staining intensity measurement following low stretch, N = 4 (**B**) or high stretch, N = 5–8 (**C**). 3D image from hSVECs stained with phalloidin at static (Z dimension: 6 µm) or high stretch (Z dimension: 9 µm) for 48 h (**D**). The F-actin reduction in hSVECs submitted to 48 h of high stretch is reversible after 24 h of stretch cessation as illustrated by confocal image (**E**) and staining intensity measurement, N = 3 (**F**). Confocal images of F-actin stained with phalloidin (red) from HAEC submitted to high stretch for the indicated time (**G**) and its staining intensity measurement, N = 3 (**H**). All confocal representative images stained for nucleus with DAPI (blue), magnification: ×400, scale (white bar): 20 μm (**E**) or 50 μm (**A,G**). All stretched-cells have their own static control done in the same time (24 h and 48 h). Since the static groups presented the same pattern regardless the period, only one static control was selected to be represented in the panels. Staining measurement was done through ImageJ software. The data are represented as mean ± SEM of at least 3 independent experiments with cells obtained from different donors. One-way ANOVA with Tukey's multiple comparisons test **(B,C,H)** and unpaired t-test **(F)** was used for statistical analysis,* indicates p < 0.05 and ** indicates p < 0.01.

hSVECs cultured under LS displayed a pattern and intensity of cortical F-actin similar to static conditions (Fig. 3A,B). In contrast, 24 h HS resulted in significant formation of actin stress fibers (Fig. 3A) and a marked reduction in F-actin staining by 48 h (Fig. 3A,C). 3D reconstruction of z-stack slices confirmed a decrease in F-actin volume throughout the cell (Fig. 3D). This phenomenon was reversible, with the reappearance of actin stress fibers 24 h after cells were placed back under static conditions (Fig. 3E,F). The F-actin reduction was only observed in hSVECs; HAECs showed no changes in actin fiber architecture irrespective of stretching conditions (Fig. 3G,H).

F-actin formation relies on the availability of monomeric G-actin^18^. Surprisingly, we found no difference in G-actin intensity of stretched hSVECs compared to static cells, although the staining and 3D reconstruction suggested a time-dependent pattern indicating cytoplasmic-nuclear shuttling along the time exposed to HS (See Suplementary Fig. S3A-D online). Cofilin, one of the proteins that mediates G-actin entry into the nucleus^19,20^, also showed similar behavior with no staining changes compared to static condition, but displayed significant nucleocytoplasmic shuttling in response to HS (See Suplementary Fig S3E-G online).

**Figure 4:**
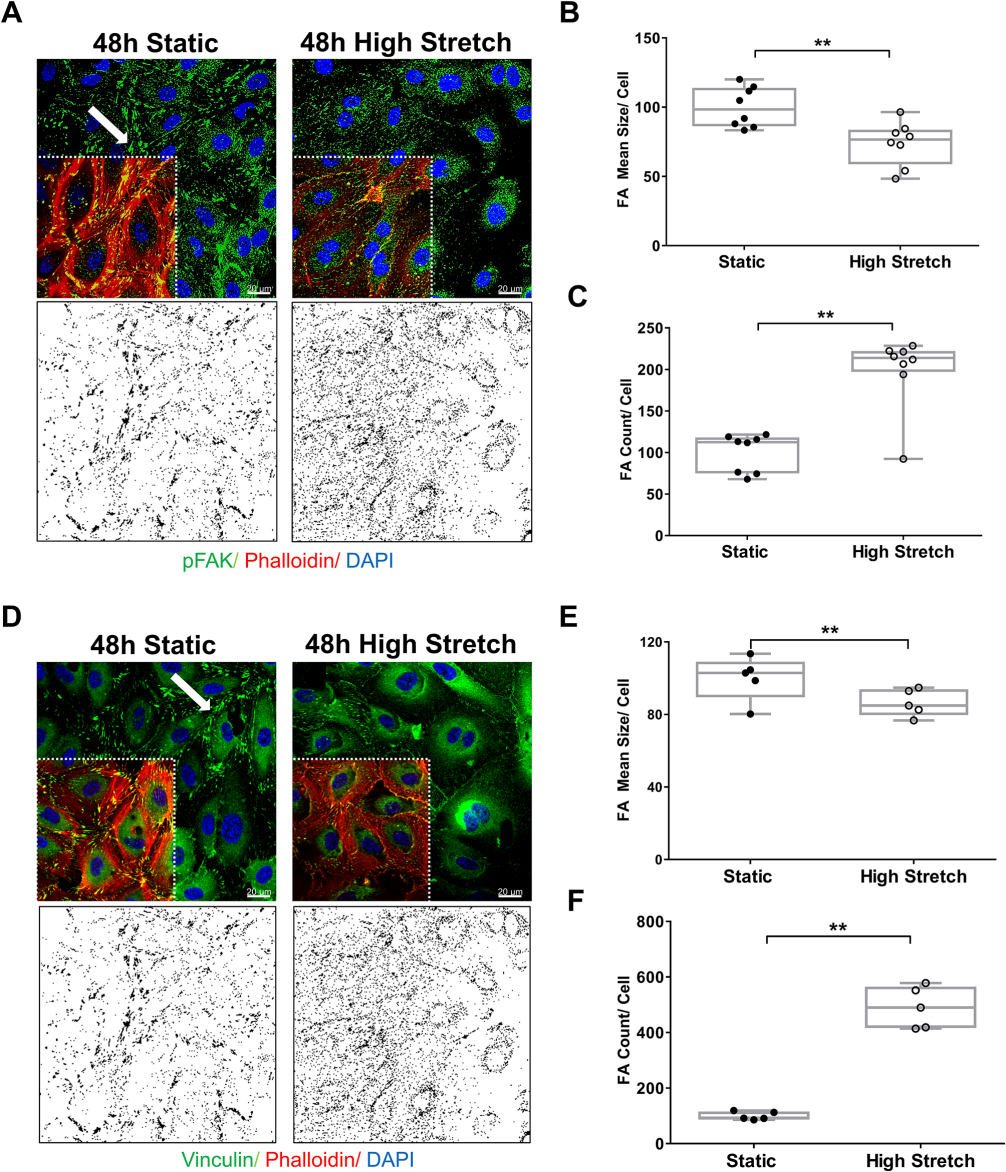
Sustained high stretch on hSVECs modifies focal adhesion (FA) activity. Confocal images of FA stained for pFAK (**A**, green) and vinculin (**D**, green) after 48 h of high stretch. The cells were also co-stained for F-actin (red) and nucleus (blue). Magnification: ×400, scale: 20 μm (white bar). The mask generated for analysis by IMAGE J illustrates the selected regions used for measurement (**A,B**, bottom panel). FA mean size (**B,E**) and FA count (**C,F**) was quantified by pFAK and vinculin markers, respectively, and normalized by numbers of nucleus. All measurements were performed using ImageJ software. The data are represented as mean ± SEM of at least 2 independent experiments with cells obtained from different donors. Unpaired t-test was used for statistical analysis, ** indicates p < 0.01.

Due to F-actin reduction, we evaluated focal adhesion (FA), an important player in mechanotransduction^21,22^. FA complexes connect to the actin cytoskeleton and drive their stabilization, while the cytoskeleton regulates FA activity (phosphorylated FA kinase, FAK), size and maturation^21,22^. Therefore, we assessed FA using p-FAK^Y397^ and vinculin staining to measure FA features (Fig. 4). Static hSVECs displayed fusiform FAs concentrated at the cell border (highlighted by the arrow in Fig. 4A,D), while sustained stretch induced more widely distributed and punctate staining of FAs (Fig. 4A,D). The mask generated for analysis by IMAGE J, illustrates the selected regions used for quantification (Fig. 4A,D; bottom panel). The measurements show that static cells displayed increased FA mean size compared to 48 h high-stretched hSVECs, as evidenced by both FA markers used (*p-FAK*^*Y397*^*and vinculin*) (Fig. 4B,E). In contrast, the number of FAs was reduced in the static group compared to the HS group (Fig. 4C,F).

### HS increases oxidative stress and NAC treatment diminishes stretch-induced endothelial dysfunction

**Figure 5:**
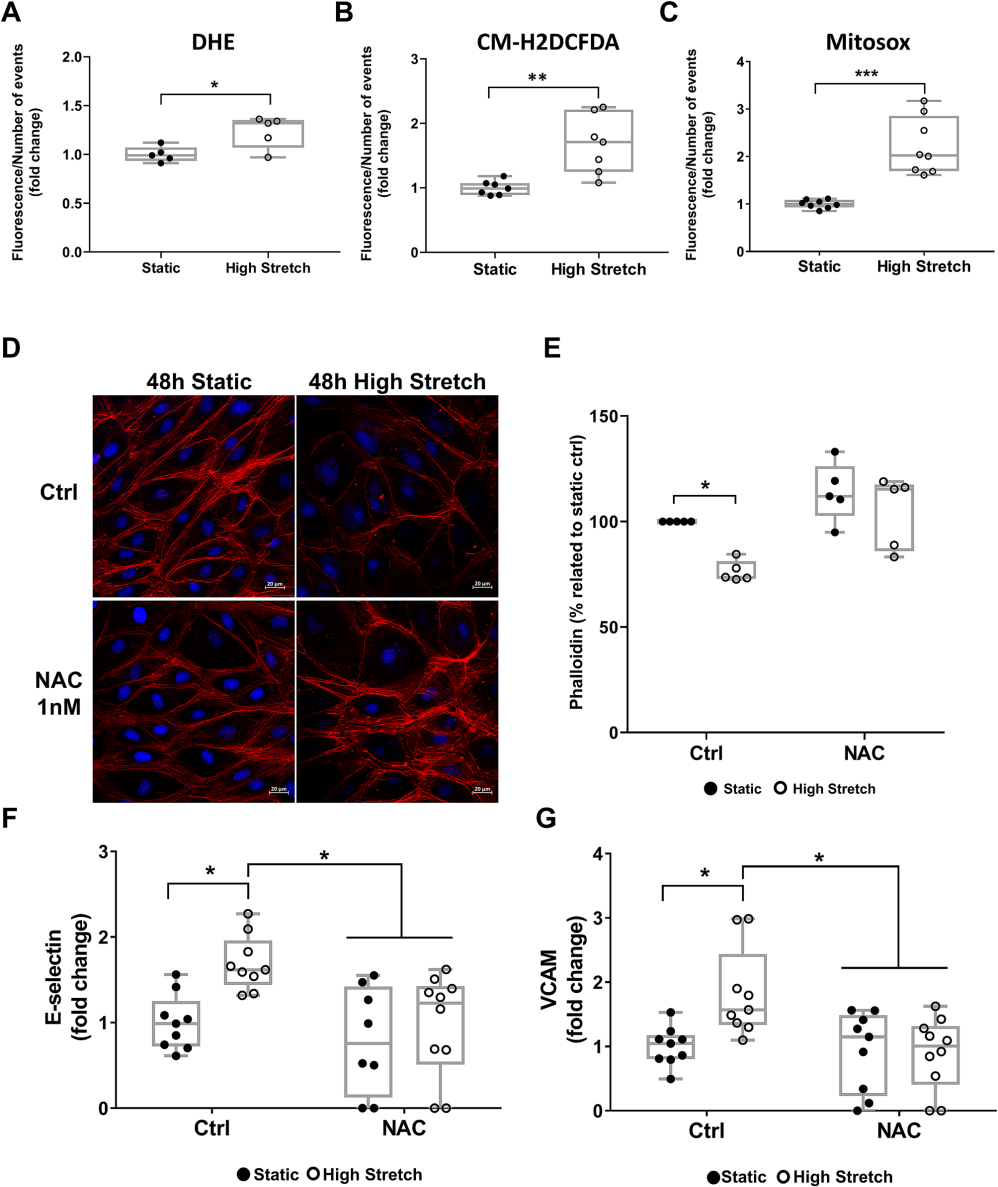
ROS induced by high stretch leads to F-actin remodeling and endothelial dysfunction. The production of ROS in hSVECs submitted to 48 h of high stretch was measured by DHE N = 5 (**A**), CM-H2DCFDA N = 7 of 2 independent experiments (**B**), and Mitosox N = 8 of 2 independent experiments (**C**). Confocal images (**D**) and intensity measurement (**E**) of F-actin (red) after 48 h of high stretch with or without the antioxidant NAC 1 nM, N = 5. Nucleus with DAPI (blue), magnification: 400x, scale: 20 μm (white bar). Flow cytometry analysis for E-selectin N = 9–10 (**F**) and VCAM1 N = 9–10 (**G**) in hSVEC stimulated with high stretch for 48 h with or without the antioxidant NAC (the data from Fig. 1C,D are re-plotted here to better observation of the effect of NAC, all experimental groups presented were done in the same time). The data are represented as mean ± SEM of at least 2 independent experiments with cells obtained from different donors. Unpaired t test was **(A–C)**, two-way ANOVA with Sidak's multiple comparisons test **(E)**, and Tukey's multiple comparisons test **(F,G)** was used for statistical analysis, * indicates p < 0.05. Flow cytometry data are plotted as fold change related to static control group and representative flow cytometry plots are in the Supplementary Information.

It is known that pathological mechanical stretch induces excessive ROS in cultured ECs^23–25^. Accordingly, we observed that total intracellular and mitochondrial ROS increase in hSVECs after 48 h of HS (Fig. 5A–C). We hypothesized that HS-induced ROS is one of the underlying mechanisms associated to cytoskeleton remodeling and endothelial dysfunction. We tested this idea by treating the cells with the antioxidant NAC and observed a decrease in HS-induced cytoskeleton actin depolymerization (Fig. 5D,E). Moreover, the stretch-induced increase in the inflammation markers E-Selectin and VCAM in hSVECs (Fig. 1C,D) was also diminished by NAC (Fig. 5F,G). These data is consistent with the idea that ROS plays an important role in saphenous EC dysfunction elicited by HS.

## Discussion

Here, we provide evidence that sustained HS is pathological for hSVECs, resulting in proinflammatory phenotype with no evidence of endothelial-to-mesenchymal transition. The venous EC dysfunction involves cytoskeleton actin fiber remodeling which is, at least in part, mediated by ROS.

The importance of physical forces, notably shear-stress and stretch, for vascular mechanotransduction is well recognized. Previously, we demonstrated a two-fold increase in NO release by hSVECs exposed to shear stress compared to static condition^26,27^. In the present work, we expanded these findings and demonstrated an intensity dependent effect of stretch on NO reduction by hSVEC. The outcomes of vascular grafting appear to be associated with this phenotype since NO release is increased in mammary artery grafts that display greater performance compared to SV grafts. Moreover, NO impairment has been demonstrated to induce neointima hyperplasia in vein grafts^28,29^. Endothelial nitric oxide synthase (eNOS) is the main enzyme responsible for NO production in ECs, and the actin cytoskeleton is one of the regulators of eNOS activity^30^. eNOS binds both G-actin and cortical F-actin, and this association directly interferes with its activity in ECs^30^. One may speculate that the changes in F-actin remodeling, as observed along HS, affect eNOS activity and contribute to endothelial dysfunction. Another scenario is the reduction of NO bioavailability as a consequence of eNOS uncoupling, which produces superoxide anion (O^2−^) instead NO^31^, that could also contribute to the increased ROS content we observed in hSVECs under sustained HS. Finally, sustained HS might also induce epigenetic changes which could modulate gene expression and contribute to the EC phenotype changes and development of vascular disease^32,33^. The interplay between cyclic stretch and epigenetic mechanisms is largely unexplored and deserves to be investigated in future studies.

The HS-induced EC dysfunction phenotype was further corroborated by the increased expression of the inflammatory markers E-selectin and VCAM 1 and THP1 adhesion in hSVECs. Our data are in line with results obtained in tissue analysis, where expression of VCAM 1, ICAM 1 and P-selectin has been previously demonstrated in SVs exposed to high pressure^9,34^. The EC dysfunction has also been associated with the adoption of a mesenchymal phenotype^15,16,35,36^ and neointima development in vein graft^17,37,38^, but we failed to observe neither the induction nor the potentiation of EndMT in hSVECs exposed to sustained HS. Interestingly, mechanical stretching has been shown to induce EndMT in human umbilical vein endothelial cells (HUVECs) and primary mouse lung ECs^17,39–41^, which could indicate differences to vein tissue heterogeneity, although we showed that hSVECs are not resistant to this process, since they underwent EndMT upon treatment with classical biochemical inducers (TGF-β_2_/IL-1β). At present, this discrepancy cannot be accounted and additional data shall clarify the context dependent features of these observations. For instance, HUVECs differ from venous ECs in adult tissue, since the umbilical vein transports oxygenated and nutrient-rich blood to the fetus, with a greater volume and flow than other veins^42^. Additionally, ECs from the lungs are constantly exposed to higher levels of oxygen and mechanical stimuli than are hSVECs^25,43,44^. Finally, the primary cells used here were obtained from older adults (60–80 years) who underwent revascularization. Isolated HS did not induce EndMT even in an inflammatory context, however we cannot rule out its involvement together with disturbed flow, since all hemodynamic forces are altered during vein graft.

Together with EC dysfunction phenotype, we observed a marked reduction in phalloidin staining indicating actin cytoskeleton remodeling under prolonged HS. The disappearance in F-actin staining was restricted to sustained HS applied to hSVECs, while arterial endothelial cells (HAECs) did not change their phenotype. Changes in ECs actin pattern and cell shape were previously exhibited after ex vivo perfusion of saphenous vein in arterial conditions^45^. En face microscopy evidenced that SV endothelium exposed to venous condition displays cortical pattern without altered the cell shape, whereas arterial regimen lead to reduced cortical staining and increased stress fibers after 20 h^45^. These data corroborate our in vitro results obtained after 24 h of HS, and it will be important to assess the effect of this mechanical stimuli on SV or hSVECs for long periods of time. Previous studies that varied long-term multiaxial stretch strain up to 96 h in pulmonary artery endothelial cells (PAECs) did not show changes in the actin cytoskeleton pattern, but it is important to emphasize that the pulmonary microenvironment and PAEC behavior in response to several stimuli are somewhat unique compared to other ECs^25,43,44^. Yet, there was a difference in the F-actin pattern when PAECs preconditioned with HS (18%) were exposed to chemical treatment, such as thrombin treatment, namely, increased barrier disruption and EC permeability^25,43,44^. One may speculate that cytoskeleton actin remodeling here described is part of a “cell panic button” since venous cells are not programmed or prepared to deal with the magnitude of the arterial physical forces. These stimuli may simply exceed the capacity of the counter regulatory processes, requiring a sort of cell resetting prior to respond to outside stimuli again. Another scenario is that stretch modifies F-actin conformation and, as a result, phalloidin can no longer bind it. We found no difference in F-actin:G-actin ratio between static and stretched hSVEC (Supplementary Fig. S4 online) and recent publications showed that different F-actin markers (pholloidin, LifeAct) binds to specific regions of F-actin^46^. It has also been demonstrated that actin-binding domains of accessory proteins are sensitive to F-actin conformation^47^, and stretch may be changing the binding of accessory proteins to change EC function and phenotype.

Actin remodeling is a dynamic process that involves many proteins, such as G-actin and cofilin. Our results are consistent with a time-dependent dynamic nuclear shuttling of G-actin in hSVEC exposed to HS. The nuclear G-actin influences gene expression via a variety of mechanisms to influence cellular function^48^. For instance, actin accumulation in the nucleus was proved to change the cytoskeleton and FAs in keratinocytes, impairing the cell motility^49^. As G-actin lacks a nuclear localization sequence (NLS)^19,20^, its translocation to the nucleus occurs through another partner. Cofilin is a member of actin-binding proteins family that upon phosphorylation facilitate the disassembly of F-actin, but is also a moonlighting protein because it can promote G actin nuclear transport^50^. Total cofilin displayed similar time-dependent localization pattern as G-actin during HS, therefore, we speculate that mechanical stretch alters cofilin availability in different compartments of hSVECs over time to translocate G-actin and modulate the cell phenotype.

Actin cytoskeleton and FA remodeling respond to external forces and intracellular tension^21,22^. It is known that mechanical forces modify actin bundling, which in turn interferes with FA assembly^51–53^. Here, the modification of actin cytoskeleton organization occurred together with changes in FA activity upon exposure to HS. It was observed that high stretched-hSVECs showed increased number and smaller size FA indicating changes in mechanotransduction response. Shao et al. demonstrated that HUVECs with different patterns of F-actin present changes in intracellular tension and FA assembly, thus allowing the cells to be more sensitive to 10% uniaxial stretching^53^. These researchers suggested that the F-actin global architecture drives mechanotransduction and directs the cell phenotype^53^. Our data suggest that actin cytoskeleton reduction and FA remodeling may change mechanotransduction, leading to endothelial dysfunction, as illustrated by impaired NO secretion and pro-inflammatory profile.

Another mechanism associated with endothelial dysfunction is altered ROS production^24,54^, and we demonstrated that oxidative stress appears to play a key role in hSVEC dysfunction induced by HS. Mechanical stretching is known to increase ROS production in ECs^23,24^, and in fibroblasts, this is accompanied by F-actin fragmentation^55^. Cytoskeletal proteins and proteins related to their regulation are highly susceptible to oxidation, given the large amount of cysteine reactive residues^56^. The impact of ROS on ECs from venous origin can be more pronounced, as they are more sensitive to ROS effects than ECs from arterial sources^57^. Furthermore, there is evidence that the oxidative capacity of saphenous vein is reduced in older compared to younger people^58^. Notably, combined antioxidant treatment prevented adhesion molecule activation and actin filament depolymerization in hSVECs, and might represent potential intervention for SV graft failure. Despite the fact of the limitations of the in vitro approach, there is a large body of evidence showing that oxidative stress modulation is critical for neointima formation in vein grafts^59–61^.

Considering the present data and evidence that the manipulation of SV before the surgery increases ROS, and may contribute to vein hyperplasia in long term organ culture preparations^62^, or measures to prevent excessive intraluminal pressure distension during graft handling that reduces the neointima formation^63^, one may consider ROS a plausible candidate to be targeted under the setting of the revascularization surgery to improve outcomes.

## Conclusion

Altogether, we provided evidence that increased oxidative stress and actin cytoskeleton remodeling play a role in HS-induced saphenous vein endothelial cell dysfunction. It is tempting to speculate that this pattern of endothelia dysfunction and cell adaptation described contribute to predispose saphenous vein graft to failure.

## Methods

### Cell culture

#### Endothelial cells

hSVECs were isolated from fragments of human SVs obtained from patients undergoing aortocoronary bypass surgery at Heart Institute (InCor) University of São Paulo Medical School; as previously described^26,27,64^. All individuals gave informed consent to participate in the study, which was reviewed and approved by the local Ethics Committee—Comissão de Ética para Análise de Projetos de Pesquisa do HCFMUSP (SDC 4292/15/119, CAPPesq153/15). All methods were carried out in accordance with the guidelines of the National Council of Ethics in Research, resolution 466, which abide to the World Medical Association Declaration of Helsinque—ethical principles for medical research involving human subjects.

hSVEC were isolated by incubating vein segments with 1 mg/mL collagenase type II for 1 h at 37 °C. After washing with phosphate buffer solution (PBS), the cell pellet was resuspended and cultivated in EGM-2 medium (Lonza) with 2% fetal bovine serum (FBS). ECs were characterized by their cobblestone appearance and by positive staining for VE-cadherin and PECAM-1. All experiments were performed with hSVECs at passages 4–8. Commercially available human aortic endothelial cells (HAECs) were purchased from Lonza and used between passages 4–8 with EGM2 + 2% FBS.

Endothelial-to-mesenchymal transition (EndMT) chemical induction: hSVECs at 100% confluence were treated with TGF-β_2_ (10 ng/mL) and IL-1β (10 ng/mL), as previously described^65^, for 4 days. The medium was changed every day, and hSVECs were harvested for the protein or staining protocol.

#### Monocytes cells

THP-1 cells (human acute monocytic leukemia cell line) were cultured in RPMI medium containing 10% FBS, 100 U/mL penicillin, 100 mg/mL streptomycin and beta-mercaptoethanol 0.05 mM. Cells were maintained in log phase and cell concentration did no exceed 1 × 10^6^ cells/mL.

### Stretch protocol

Multiaxial stretching was performed using (1) a Flexcell FX-5000TM or (2) a custom device, as previously described^66–68^. The same quantity of endothelial cells (2^10^5^ cells/well) were cultured in six-well plates with silicone membrane surfaces coated with collagen type I prior to cell seeding. 100% confluent cells were exposed to low (2.5–5%) or high (12.5–15%) levels of multidirectional stretch to mimic venous or arterial conditions, respectively. It is demonstrated that SV graft exposed to the arterial pulsatile pressure (120/80 mmHg) receives a circumferential stretch of 10–15%^8,10,69^. Mechanical loading was performed at a 1 Hz loading frequency for up to 48 h. Additionally, cells without mechanical loading were cultivated in the same plate type and incubator as a control group. The culture media was not changed during experimental period. Some experiments were performed with concomitant chemical treatment, with no culture media changes and daily treatments with TGF-β_2_ and IL-1β, both at 10 ng/mL. All experiments were maintained at 37 °C in humidified air with 5% CO_2_.

### Immunofluorescence staining

ECs were fixed in 4% paraformaldehyde (PFA) in PBS for 30 min at room temperature. After washing with PBS with 0.1% Tween 20 (PBST), the silicone membranes were cut into 0.5cm^2^ pieces and mounted onto culture slides. Next, the cells were permeabilized (PBS with 0.1% Nonidet P40), blocked for nonspecific binding sites (PBS with 1% BSA), and then incubated overnight at 4 °C with primary antibodies as indicated in Table 1. After the washing steps, the slides were incubated for 2 h with AlexaFluor555- or AlexaFluor 488-conjugated secondary antibodies (Molecular Probes), and nuclei were stained with diamino-2-phenylindole (DAPI) at 10 µg/µL, under conditions protected from light. Phalloidin AF 647 and G-actin AF488 (as indicated in Table 1) staining was performed for 2 h at room temperature and protected from light.

**Table 1:** Antibodies used for staining and western blot: brands, dilution and molecular weight.

Antibodies	Brand/catalog no.	Staining dilution	WB dilution	MW (KDa)
Calponin	Millipore/04-589	1:100	1:500	34
Cofilin	Abcam/ab42824	1:100	–	19
Collagen I	Abcam/ab34710	1:500	–	115
Deoxyribonuclease I (G-actin) AF488	Life/D12371	1:200	–	–
GAPDH	Abcam/ab9485	–	1:5000	37
PECAM1	Abcam/ab24590	1:100	1:1000	130
pFAK (y397)	Life/44–624	1:100	–	125
Phalloidin AF 647	Life/A12380	1:200	–	–
SM22α	Abcam/ab10135	1:100	1:1000	22
VEcad	Cell signaling/#2500	1:100	1:1000	115
Vinculin	Sigma/V9131	1:100	-	116

Stained samples were imaged using a laser scanning microscope 510 (Carl Zeiss) or an Olympus confocal microscope. All laser parameters were established using unstained (negative control using only secondary antibody) and static controls. The set up was maintained throughout all experimental samples. For each experiment, 5 to 10 images at the same focal plane were taken for further quantification. In some cases, Z-stack slices were made for 3D reconstruction. ImageJ software was used to quantify all samples, including FA count and size (https://imagej.nih.gov/ij/). For measurement purpose the areas for quantification were randomly selected, and the intensity were normalized by the number of nucleus. We quantified the nuclei for each image and observed similar number of cells in static and stretched groups, as demonstrated in the Supplementary Table S1 online.

### Western blot

Cells were washed with PBS and lysed in SDS buffer (60 mM Tris–HCl, pH 6.8, 5% glycerol, 2% SDS). After 10 min at 95 °C, the samples were centrifuged at 10,000 xg for 10 min to remove cellular debris. The cell lysates (25 μg) were separated in SDS–polyacrylamide gels, and the proteins were electrotransferred to PVDF membranes (Millipore, Billerica, MA). The membranes were incubated for 1 h in blocking buffer (5% bovine serum albumin (BSA), 10 mM Tris–HCl, pH 7.6, 150 mM NaCl, and 0.1% Tween 20) at room temperature and then probed with primary antibodies. Each membrane was incubated overnight with specific primary antibodies, as demonstrated in Table 1. After HRP-conjugated secondary antibody incubation, signal detection was performed with enhanced chemiluminescence reagents (GE Healthcare, Sweden) and the LAS-4000 system (Fujifilm). GAPDH protein levels were used to normalize the results, and the bands were quantified by ImageJ software (https://imagej.nih.gov/ij/).

### Nitric oxide (NO) detection

To estimate NO release, conditioned media was used to measure nitrite (NO_2_) accumulation. A sample from the conditioned media and reducing solution (potassium iodide/acetic acid) was analysed using a Sievers TM-280 nitric oxide analyzer (NOA, Sievers Instruments Inc., Boulder, CO, USA) according to the manufacturer's protocol. Concentrations were based on previous standard curve calibration (sodium nitrite at 0.05–20 µM), and the values were normalized by total volume and protein concentration measured with a BCA protein assay kit (Pierce Biotechnology), following the manufacturer’s instructions.

### Flow cytometry

Human SVECs were exposed to 48 h of high stretching with or without the presence of the antioxidant NAC (1 nM). Cells were then labelled according to the BD Bioscience flow cytometry staining kit protocol (BD 562725). Briefly, cells were harvested, and the supernatant was removed. Afterwards, the samples were fixed and permeabilized for 40 min. The cells were then vortexed and centrifuged, and the supernatant was removed. Next, the cells were treated with VCAM- or E-selectin-conjugated fluorescent antibodies for 50 min in a light-protected environment. Afterwards, the cells were again vortexed and centrifuged, and the supernatant was removed. Subsequent fluorescent measurements were made using BD LSR II Fortessaflow cytometer (BD Biosciences) with at least 10,000 events measured per sample. Further analysis and quantification were performed using FlowJo software.

### Monocyte adhesion assay

THP1 cells were fluorescently labeled with Calcein AM (5 µM, Thermo Fisher Scientific) for 30 min at 37 °C. Labeled cells were added at 1 × 10^6^ cells/well on top of the hSVEC submitted for either 48 h of high stretch or the static control group, and incubated for 1 h at 37 °C. Thereafter, unbound monocytes were removed by gently washing (3×) with prewarmed RPMI. Adherent monocytes and hSVECs were dissociated from the culture plates using 0.05% Trypsin–EDTA, resuspended in PBS containing 2% FBS as single cells and flow cytometry-based quantification was carried out using a BD Accuri C6 Plus flow cytometer (Becton Dickinson, NJ, USA), with at least 10,000 events/ per sample. THP1 cells labeled with calcein AM showed positive fluorescence in the FL2 channel and hSVECs did not show any fluorescence. Further analysis and quantification were performed using FCS Express Flow Cytometry software.

### Reactive oxygen species measurement

Following 48 h of high stretch, hSVECs were incubated with CM-H2DCFDA (5 µM, Thermo Fisher Scientific) or DHE (5 µM, Thermo Fisher Scientific) for 30 min at 37 °C, or Mitosox (5 µM, Thermo Fisher Scientific) for 10 min at 37 °C prior to collection. The cells were washed with PBS, dissociated from the culture plates using 0.05% Trypsin–EDTA, and centrifuged at 1000*g* for 3 min. Precipitated cells were resuspended in PBS containing 2% FBS, and the fluorescence of labeled cells was monitored using a BD Accuri C6 Plus flow cytometer (Becton Dickinson, NJ, USA), with at least 20,000 events/per sample. Further analysis and quantification were performed using FCS Express Flow Cytometry software.

### F-actin and G-action ratio

The actin fractionation was performed as described in the literature. hSVEC were washed with PBS and lysed with actin stabilization buffer (0.1 M PIPES, pH 6.9, 30% glycerol, 5% DMSO, 1 mM MgSO4, 1 mM EGTA, 1% TX-100, 1 mM ATP, and protease inhibitor) on ice for 10 min. The lysate was collected and centrifuged at 4 °C for 75 min at 16.000 g. The supernatant (G-actin) was transferred to a new tube and the pellet (F-actin) was solubilized with 8 M Urea lysis buffer supplemented with phosphatase and protease inhibitor cocktails. G- and F-actin lysates were separated on 10% SDS-PAGE gel, transferred to a PVDF membrane, and blotted with a polyclonal actin antibody (Santa Cruz Biotechnology sc-1615). The bands were visualized by staining the membrane with a horseradish peroxidase-labeled anti-goat antibody for 1 h, followed by enhanced chemiluminescence. Densitometric analysis of the bands was performed using ImageJ software and the F/G-actin ratio was estimated.

### Statistical analysis

All data are shown as scatter plots with the mean ± SEM. Comparisons between 2 groups were performed using unpaired Student’s t-test. Comparisons among groups were performed using two-way analysis of variance (ANOVA) followed by Tukey´s posthoc test for comparison. Values of p < 0.05 were considered significant.

## Supplementary Information

Supplementary Information.

Supplementary Legends.

## Abbreviations

BSA: Bovine serum albumin
CABG: Coronary artery bypass graft
EC: Endothelial cell
EndMT: Endothelial to mesenchymal transition
FA: Focal adhesion
F-actin: Actin filament
FAK: Focal adhesion kinase
FBS: Fetal bovine serum
G-actin: Globular actin
HAEC: Human aortic endothelial cell
HS: High stretch
hSVEC: Human saphenous vein endothelial cell
IL-1β: Interleukin beta 1
LS: Low stretch
NAC: *N*-Acetylcysteine
NLS: Nuclear localization sequence
NO: Nitric oxide
NO_2_: Nitrite
PBS: Phosphate buffer solution
PBST: PBS with Tween 20
PECAM: Platelet endothelial cell adhesion molecule
PFA: Paraformaldehyde
ROS: Reactive oxygen species
SM22α: Smooth muscle protein 22-alpha
SMC: Smooth muscle cell
SV: Saphenous vein
TGF-β_2_: Transforming growth factor beta type 2
VCAM: Vascular cell adhesion molecule
