# Supplementary material for: High stretch induces endothelial dysfunction accompanied by oxidative stress and actin remodeling in human saphenous vein endothelial cells
Source: Sci Rep. 2021 Jun 29;11:13493. doi: 10.1038/s41598-021-93081-3 (PMC8242094; doi:10.1038/s41598-021-93081-3)

High stretch induces endothelial dysfunction accompanied by oxidative stress and actin remodeling in human saphenous vein endothelial cells

Girão-Silva T[1], Fonseca-Alaniz MH[1], Ribeiro-Silva JC[1], Lee J[2], Patil NP[2], Dallan LA[1], Baker AB[2], Harmsen MC[3], Krieger JE[1], and Miyakawa AA[1]

[1]Heart Institute (InCor), University of São Paulo Medical School, São Paulo, Brazil.

[2]Department of Biomedical Engineering; Institute for Cellular and Molecular Biology; Institute for Biomaterials, Drug Delivery and Regenerative Medicine; Institute for Computational Engineering and Sciences. University of Texas at Austin, Austin, TX, USA.

[3]Laboratory for Cardiovascular Regenerative Medicine Research Group (CAVAREM), Department of Pathology and Medical Biology, University of Groningen, University Medical Center Groningen, Groningen, the Netherlands.

Supplementary Figure S1

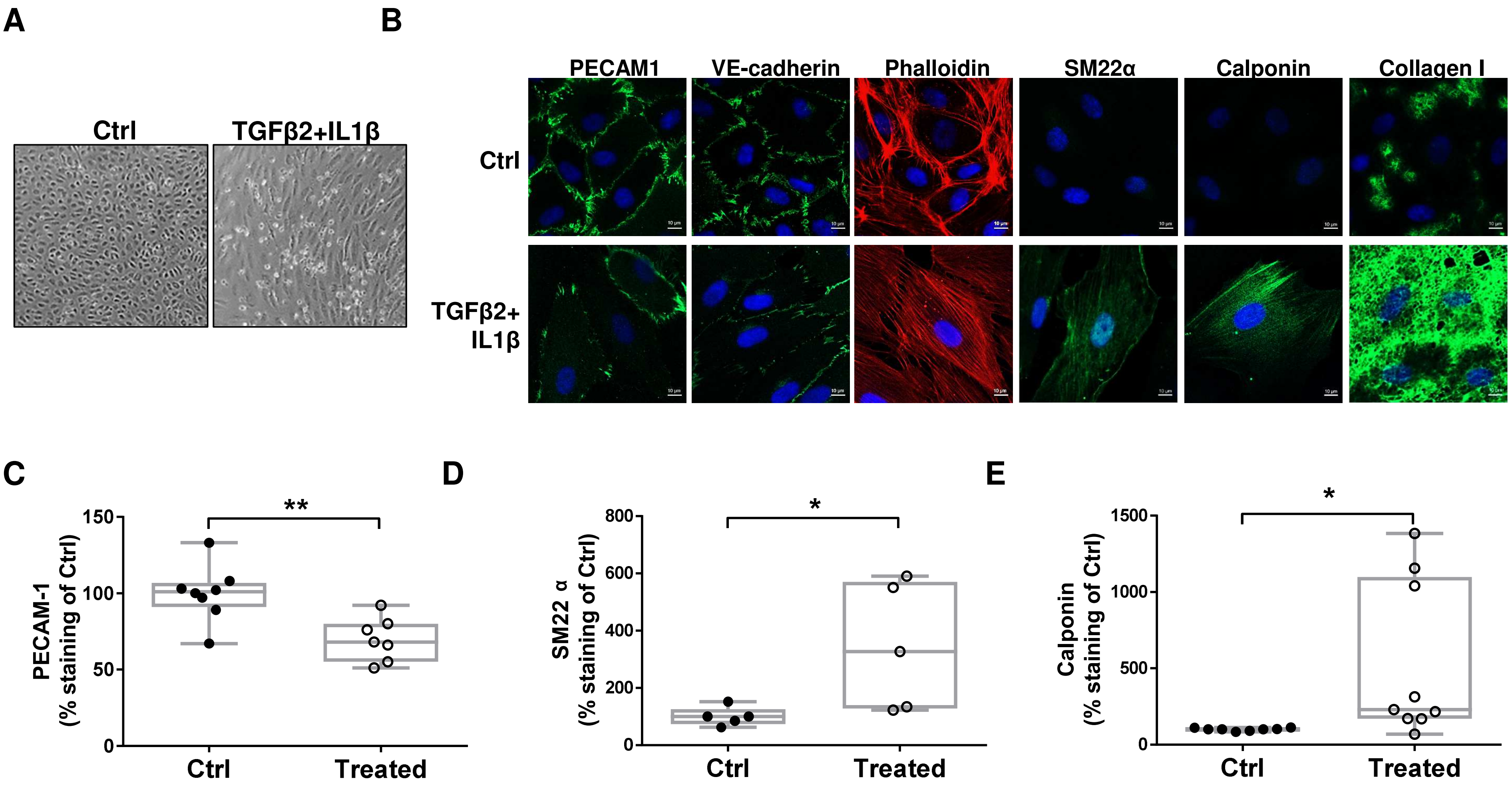

Supplementary Figure S2

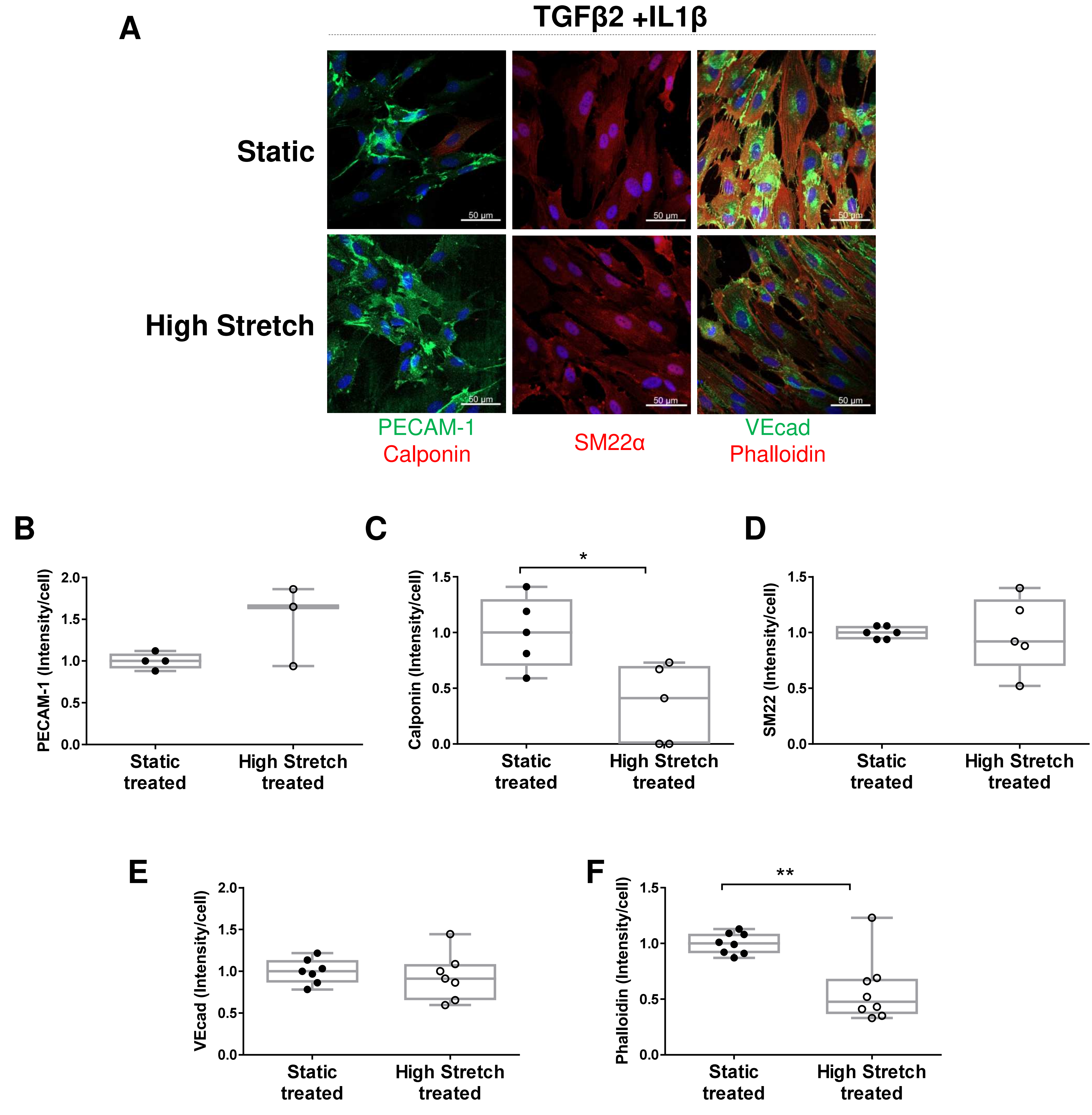

Supplementary Figure S3

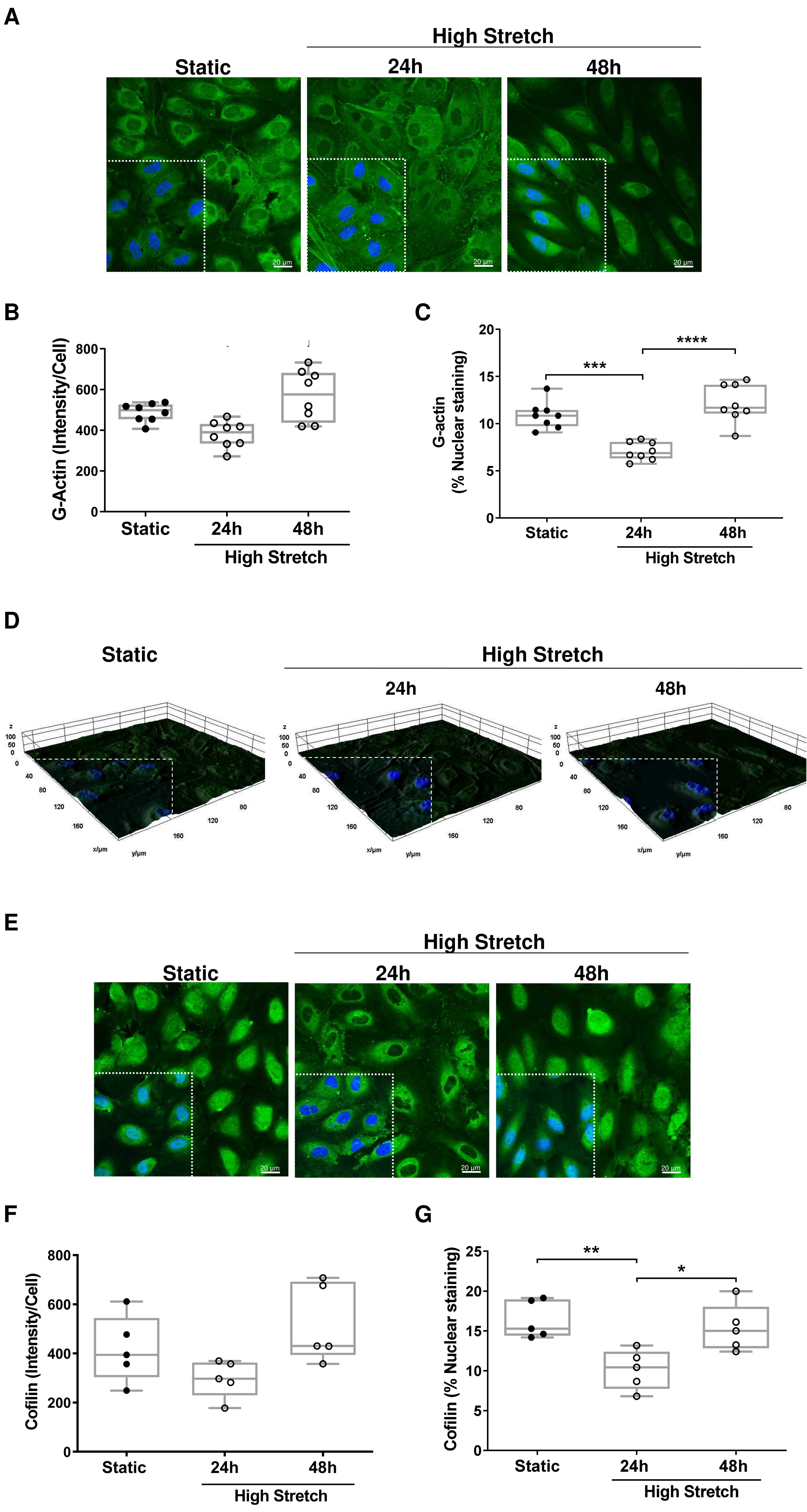

Supplementary Figure S4

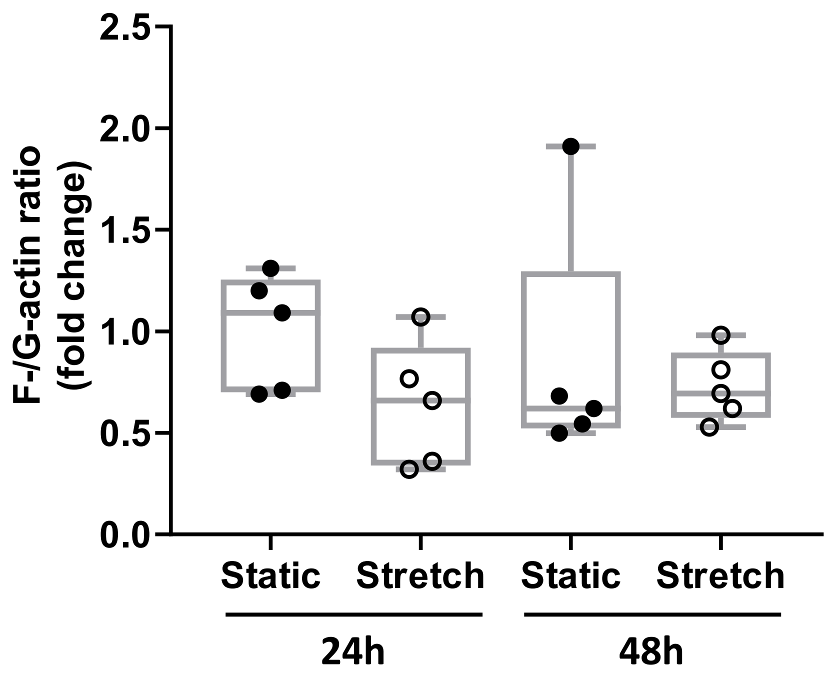

Supplementary Table S1

sample	Static 48h	Stretch 48h
1	27	21
2	22	25
3	21	23
4	25	24
5	23	20
6	26	36
7	32	41
8	42	40
Media	27,25	28,75

WB_Supplementary Information

PECAM 1

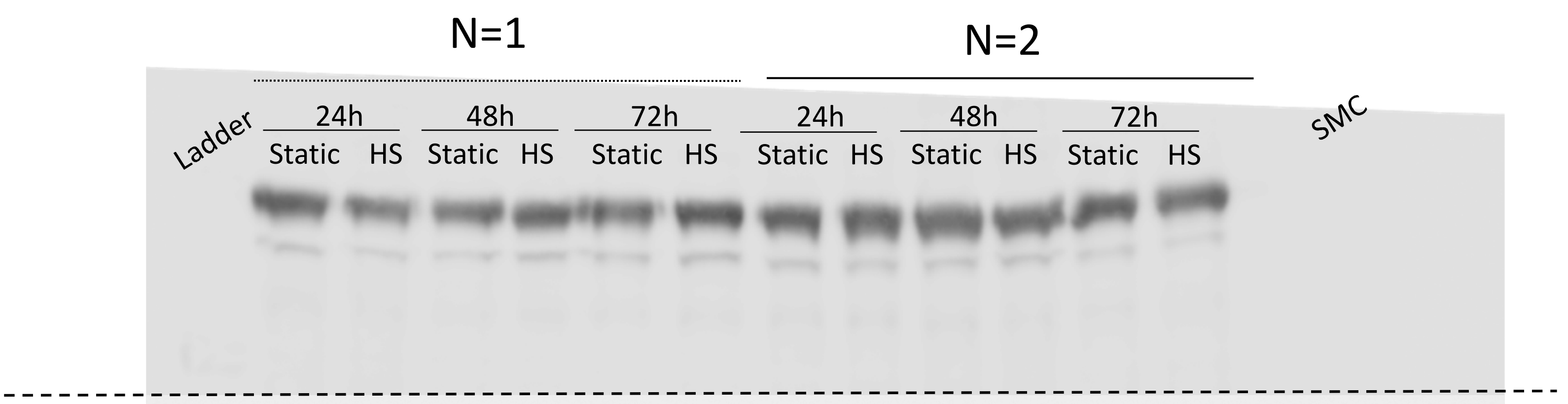

PECAM 1 + GAPDH

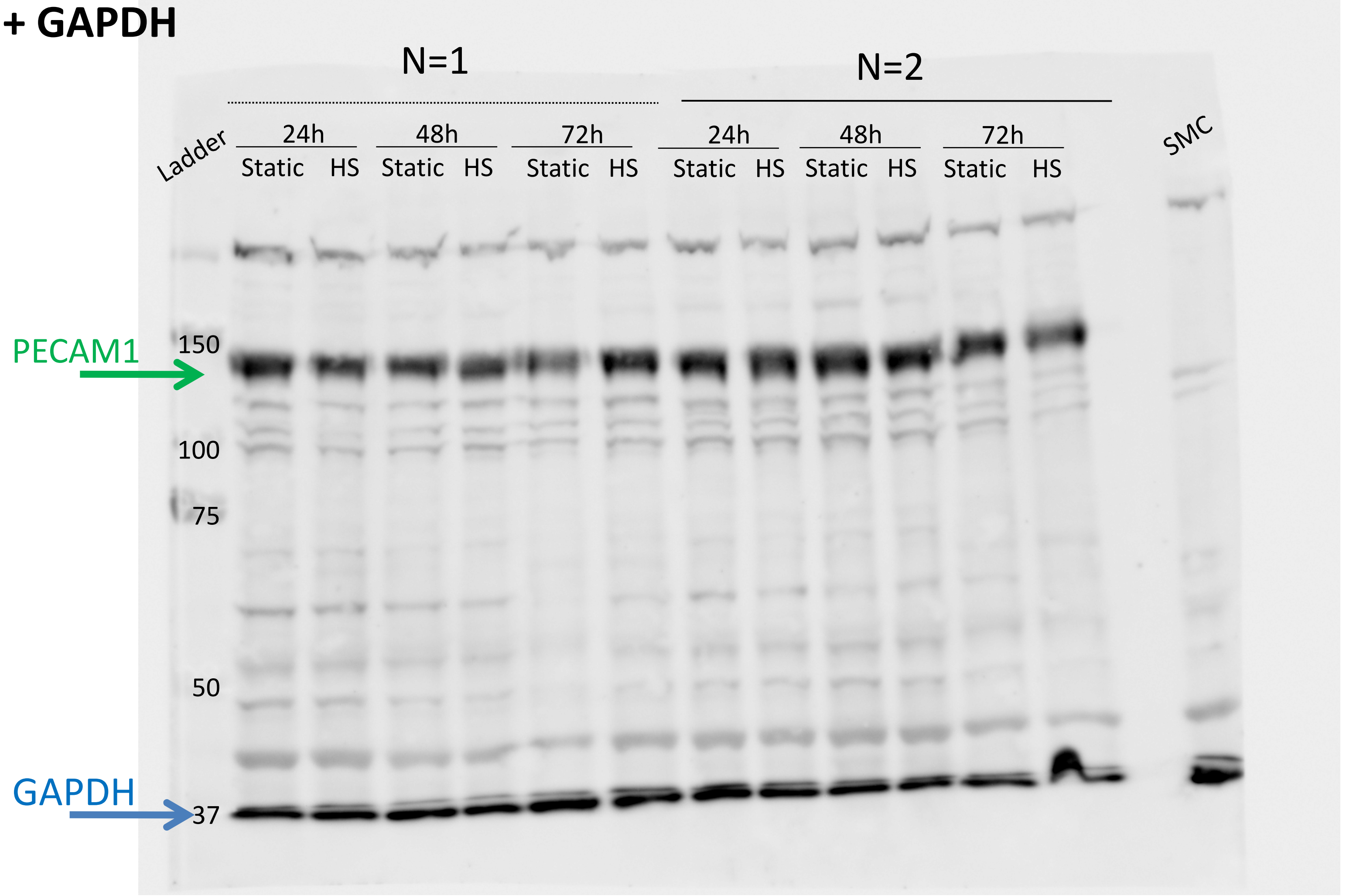

Calponin

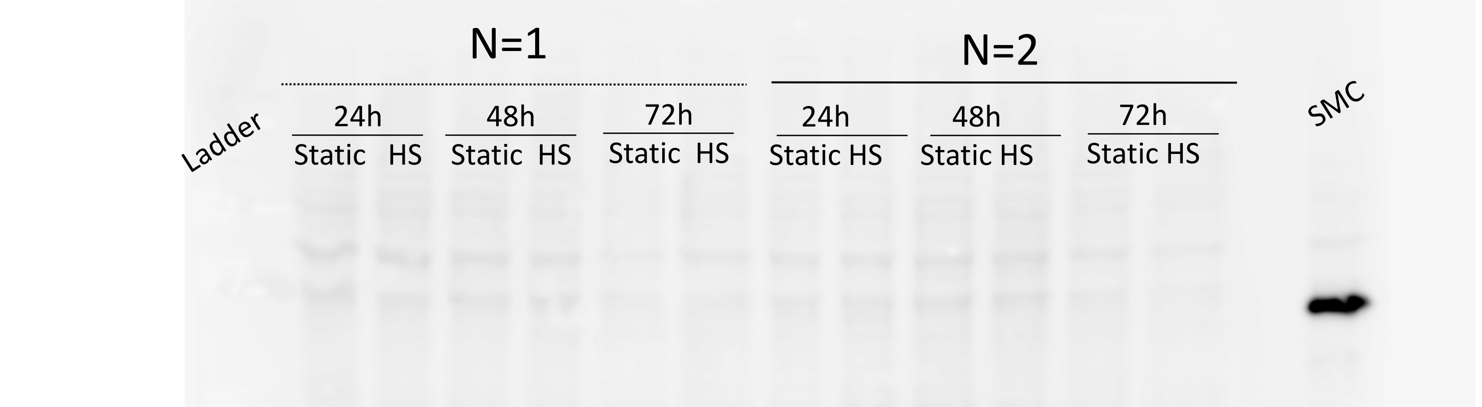

Calponin + GAPDH

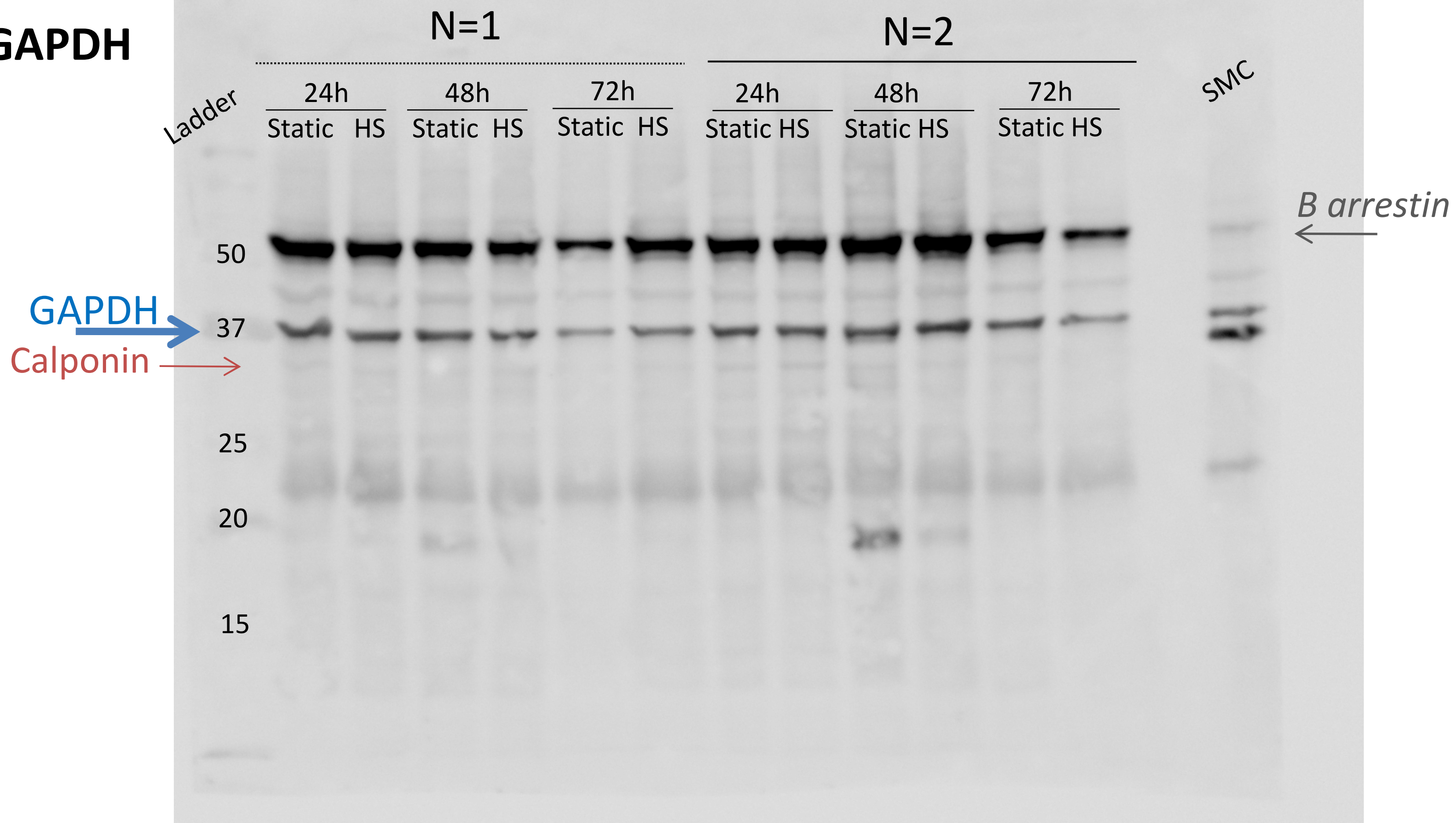

WB_Supplementary Information

SM22 + GAPDH

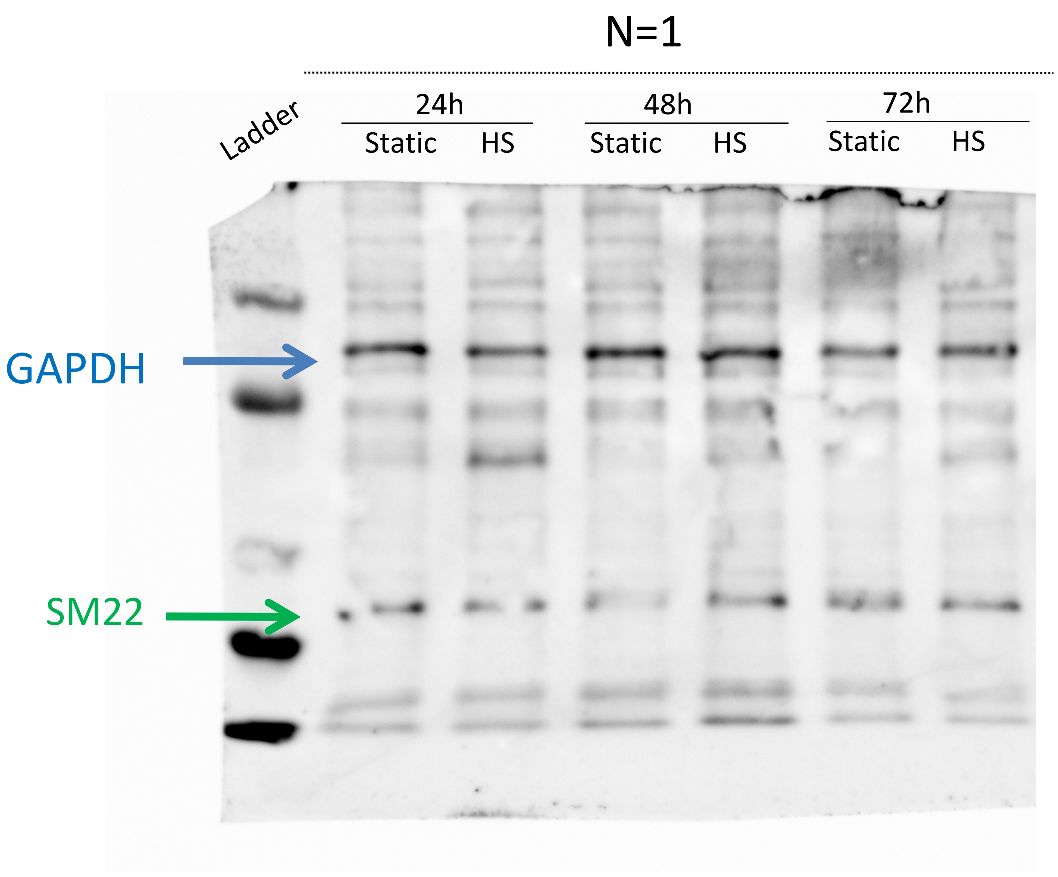

VE cadherin + GAPDH

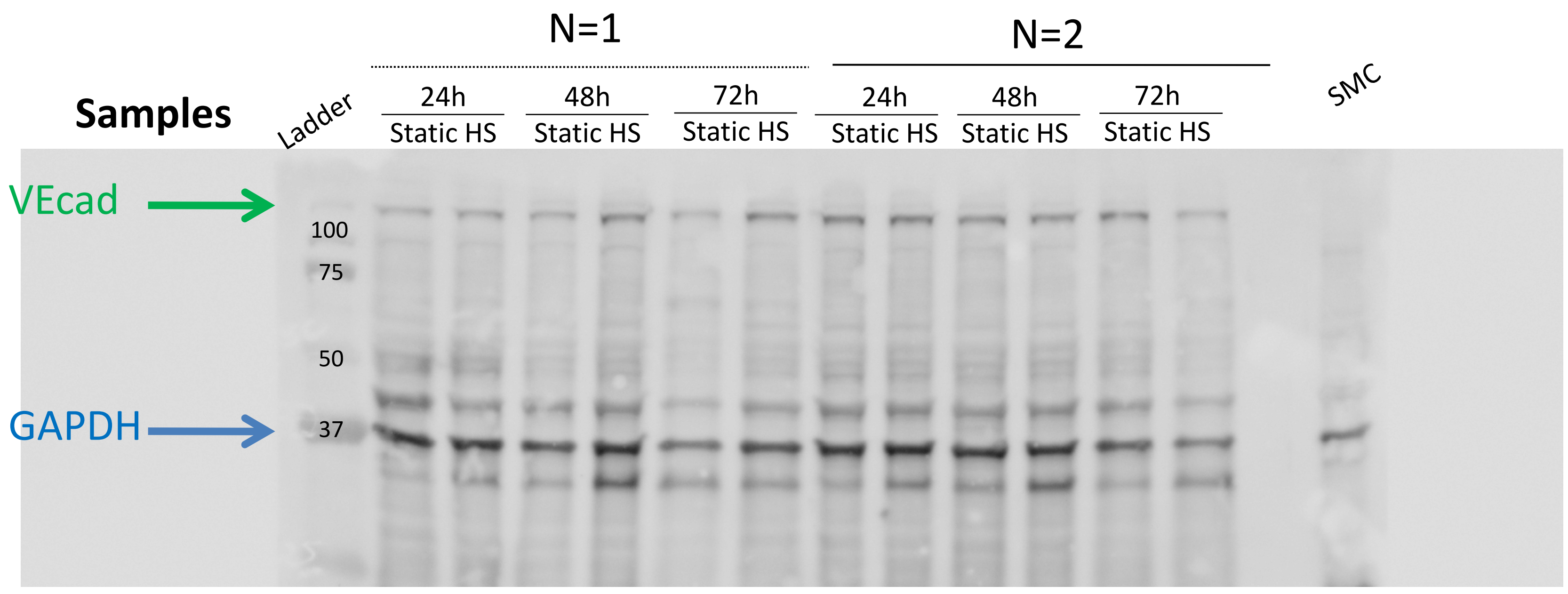

Flow Cytometry plots_Supplementary Information

Representative flow cytometry plots for data presented in Figures 1C and 1D and Figure 5F and 5G

Gated cells | E-selectin | VCAM1

A. Static

B. Static + NAC

C. High Stretch

D. High Stretch + NAC

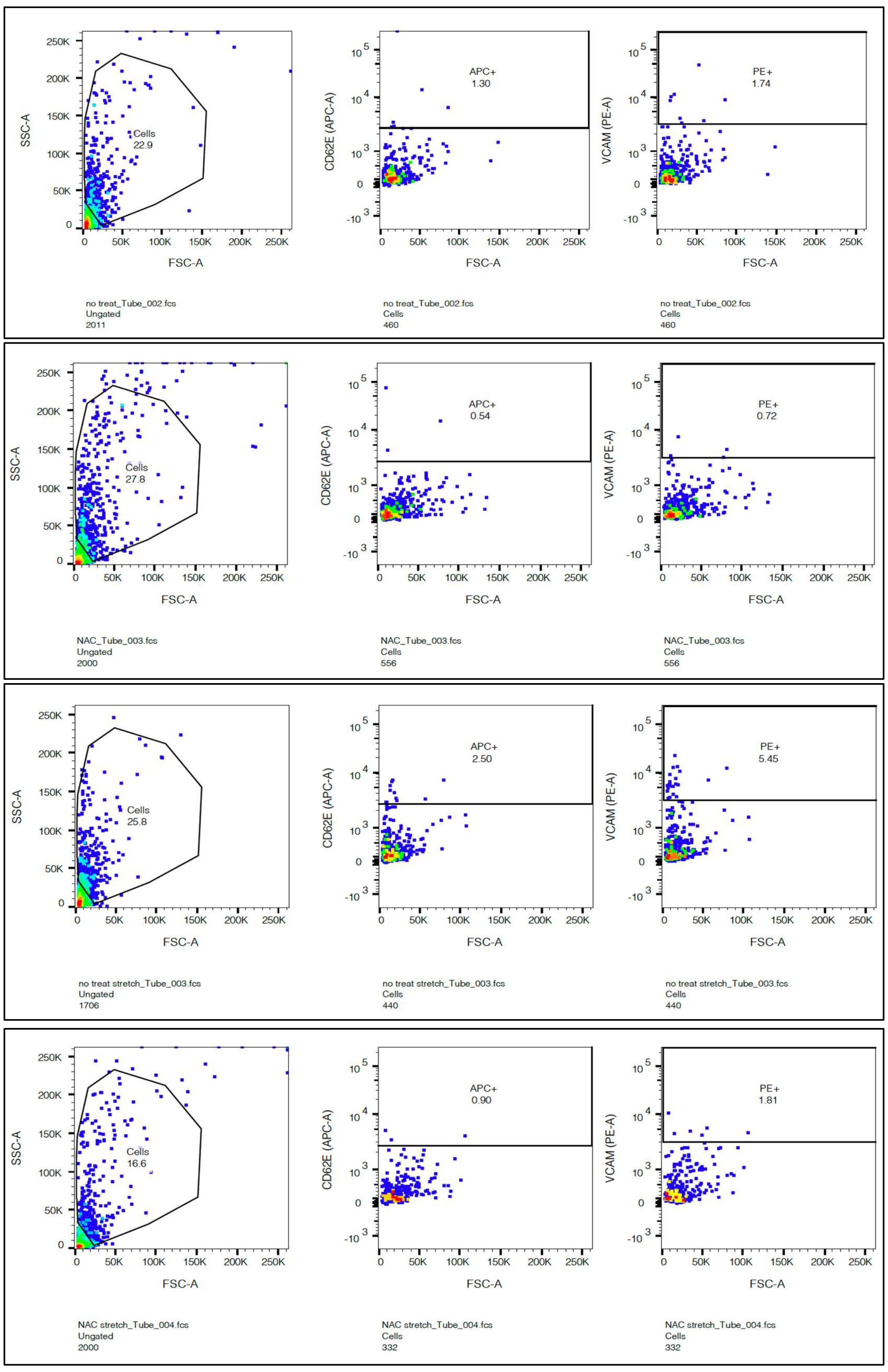

Representative flow cytometry plots for data presented in Figures 1E

Static | **High Stretch**

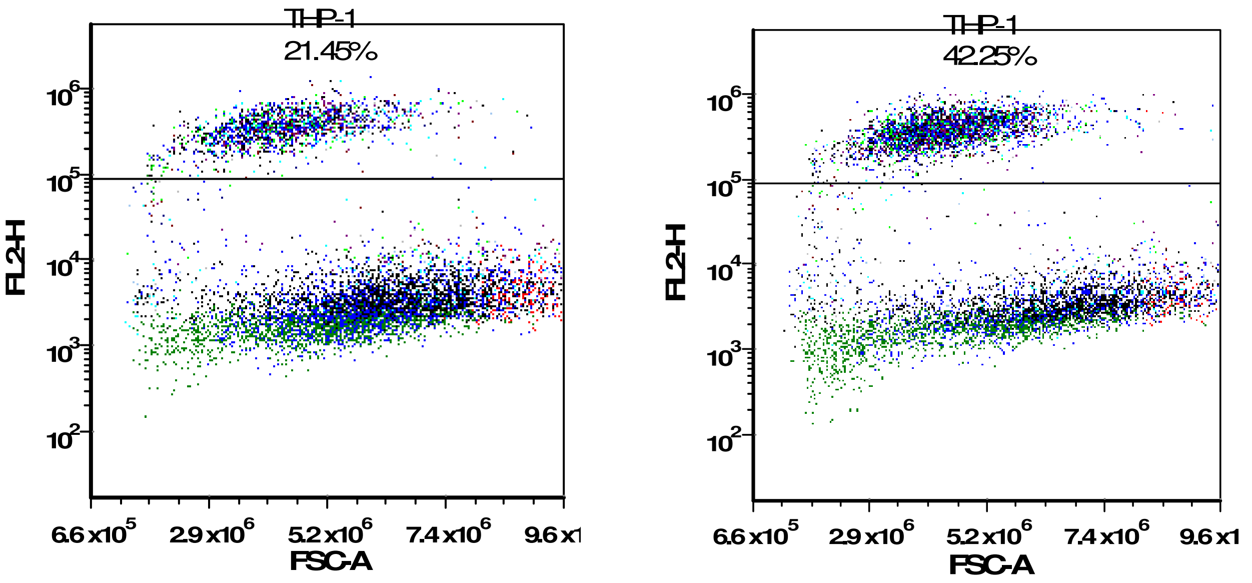

Supplement: Supplementary file 1 — Supplementary Information. [file 41598_2021_93081_MOESM1_ESM.pdf]
