# Supplementary material for: High stretch induces endothelial dysfunction accompanied by oxidative stress and actin remodeling in human saphenous vein endothelial cells
Source: Sci Rep. 2021 Jun 29;11:13493. doi: 10.1038/s41598-021-93081-3 (PMC8242094; doi:10.1038/s41598-021-93081-3)
Supplement: Supplementary file 2 — Supplementary Legends. [file 41598_2021_93081_MOESM2_ESM.docx]

**Supplementary Figure S1:** EndMT in hSVECs induced by chemical treatment. Optical (**A**) and confocal (**B**) images of hSVEC treated with TGFβ_2_ + IL1β for 4 days showed reduction in endothelial cell markers and increase in mesenchymal markers. Staining intensity measurement for PECAM1 N=4 (**C**), SM22α N=3 (**D**) and Calponin N=5 (**E**) were done by ImageJ software. Nucleus stained with DAPI (blue), scale: 10µm (white bar). The data are represented as mean ± SEM of at least 3 independent experiments with cells obtained from different donors. Unpaired t-test was used for statistical analysis, * indicates p <0.05 and ** indicates p <0.01.

**Supplementary Figure S2:** High stretch did not potentiate EndMT induced by chemical treatment. Stretched-hSVECs for 48h in the presence of TGFβ_2_ + IL1β was evaluated by immunostaining (**A**). Intensity measurement for PECAM-1 N=3 (**B**), Calponin N=5 (**C**), SM22α N=5 (**D**), VE-cadherin N=7 (**E**) and Phalloidin N=8 (**F**) were done through IMAGE J software. All confocal experiments were also stained for nucleus with DAPI (blue), magnification: 400x and scale: 50µm (white bar). The data are represented as mean ± SEM of at least 3 independent experiments with cells obtained from different donors. Unpaired t-test was used for statistical analysis, * indicates p <0.05 and ** indicates p <0.01.

**Supplementary Figure S3:** High stretch promotes dynamic changes at intracellular distribution of G-actin and cofilin in hSVEC. G-actin confocal images in green (**A**), with total staining intensity measurement (**B**) and the percentage of nuclear staining, N=3-7 (**C**). 3D image from hSVEC stained with G-actin at static (Z dimension: 6µm) or high stretch (Z dimension: 9µm) for 48h (**D**). Cofilin confocal images in green (**E**), with total staining intensity (**F**) and the percentage of nuclear staining, N=3 (**G**). All representative images stained for nucleus with DAPI (blue) as illustrated in the dashed box, magnification: 400x, scale: 20μm (white bar). All stretched-cells have their own static control done in the same time (24h and 48h). Since the static groups presented the same pattern regardless the period, only one static control was selected to be represented in the panels. Quantification of immunostaining was done by using ImageJ software. The data are represented as mean ± SEM of at least 3 independent experiments with cells obtained from different donors. One-way ANOVA with Tukey's multiple comparisons test was used for statistical analysis, * indicates p <0.05.

**Supplementary Figure S4:** High stretch does not modify F-actin and G-actin ratio. hSVEC were submitted to high stretch for 24 h or 48 h. The data are represented as mean ± SEM of 5 independent experiments with cells obtained from different donors.

**Supplementary Table S1:** High stretch doe not modify the number of cells after 48h. The number of cells were quantified in all areas selected for immunofluorescense analysis showing similar number of cells in static and stretched groups.
